# A review of neuro-ophthalmic sequelae following COVID-19 infection and vaccination

**DOI:** 10.3389/fcimb.2024.1345683

**Published:** 2024-01-17

**Authors:** Jane Shi, Helen V. Danesh-Meyer

**Affiliations:** ^1^ Faculty of Medical and Health Sciences, University of Auckland, Auckland, New Zealand; ^2^ Ophthalmology, Greenlane Clinical Centre, Te Whatu Ora – Health New Zealand, Auckland, New Zealand

**Keywords:** coronavirus, Covid-19, SARS-CoV-2, 2019-nCoV, vaccination, vaccine, immunization, neuro-ophthalmology

## Abstract

**Background:**

It has become increasingly clear that the severe acute respiratory syndrome coronavirus 2 (SARS-CoV-2) can affect most organs in the human body, including the neurologic and ophthalmic systems. Vaccination campaigns have been developed at rapid pace around the world to protect the population from the fast-mutating virus. This review seeks to summarise current knowledge of the neuro-ophthalmic manifestations of both COVID-19 infection and vaccination.

**Evidence acquisition:**

Electronic searches for published literature were conducted using EMBASE and MEDLINE on the 30^th^ of July 2023. The search strategy comprised of controlled vocabulary and free-text synonyms for the following terms in various combinations: “coronavirus, COVID-19, SARS-CoV-2, 2019-nCoV, vaccination, vaccine, immunisation and neuro-ophthalmology”. No time range limits were set for the literature search. Published English abstracts for articles written in a different language were screened if available.

**Results:**

A total of 54 case reports and case series were selected for use in the final report. 34 articles documenting neuro-ophthalmic manifestations following COVID-19 infection and 20 articles with neuro-ophthalmic complications following COVID-19 vaccination were included, comprising of 79 patients in total. The most commonly occurring condition was optic neuritis, with 25 cases following COVID-19 infection and 27 cases following vaccination against COVID-19.

**Conclusions:**

The various COVID-19 vaccines that are currently available are part of the global effort to protect the most vulnerable of the human population. The incidence of neuro-ophthalmic consequences following infection with COVID-19 is hundred-folds higher and associated with more harrowing systemic effects than vaccination against the virus.

## Introduction

SARS-CoV-2 took the world by storm at the dawn of 2020. The novel single-stranded RNA beta coronavirus affected the health and livelihoods of millions across the globe on a scale only encountered once approximately every 100 years (Wesselingh and Wesselingh, 2023). There is currently a plethora of case reports and case series of neuro-ophthalmic manifestations of COVID-19 and its vaccine counterparts (Luís et al., 2020; Betsch and Freund, 2021; Tisdale et al., 2021). As the end of this pandemic is reached, this review seeks to summarise the current knowledge of neuro-ophthalmic manifestations of both COVID-19 infection and vaccination.

## Methods of literature search

**Figure 1:**
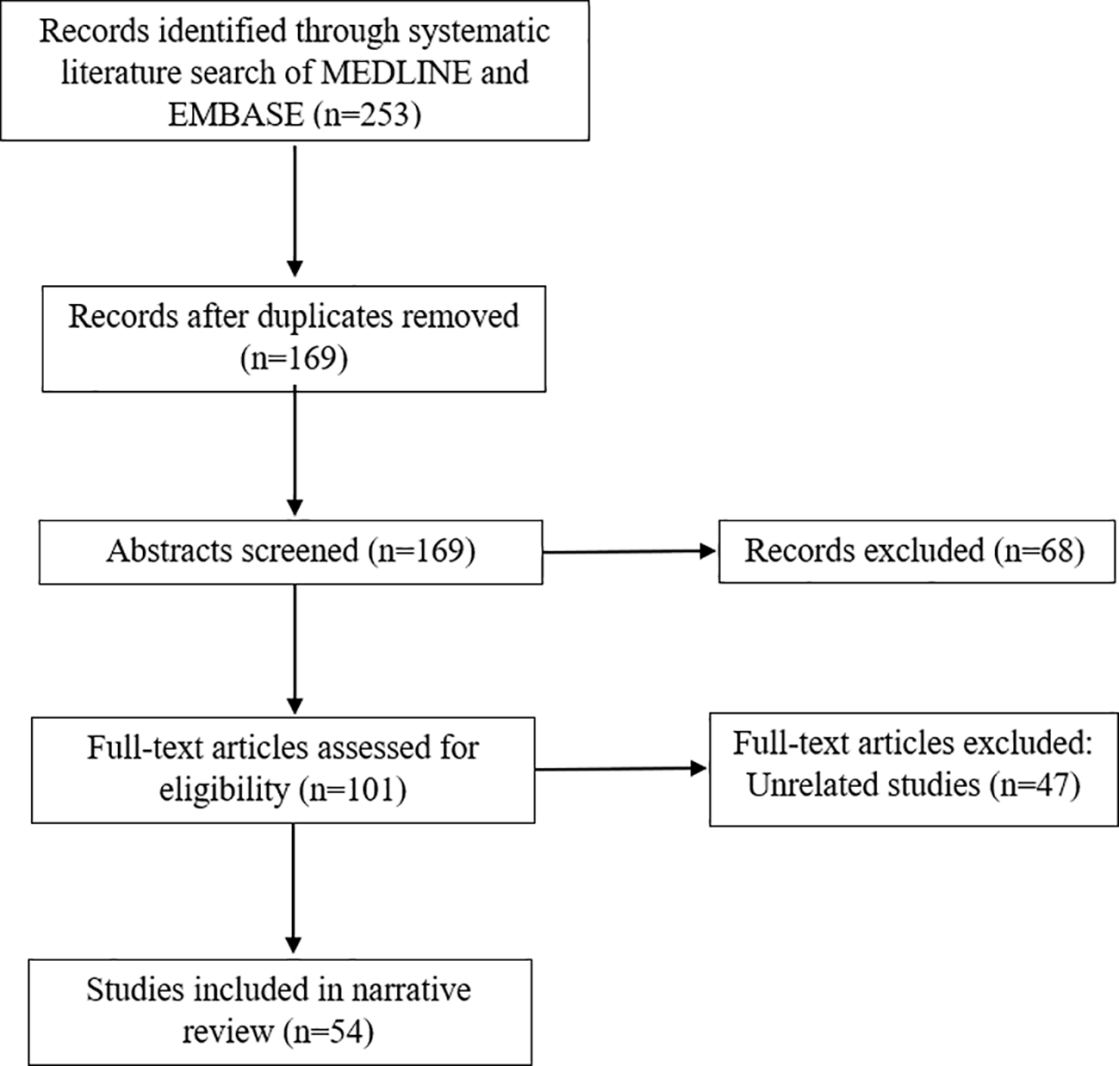
Summary of literature search.

Electronic searches for published literature were conducted using EMBASE and MEDLINE on the 30th of July 2023, see **Figure 1**. The search strategy comprised of controlled vocabulary and free-text synonyms for the following terms in various combinations: “coronavirus, COVID-19, SARS- CoV-2, 2019-nCoV, vaccination, vaccine, immunisation and neuro-ophthalmology”. No time range limits were set for the literature search. Published English abstracts for articles written in a different language were screened if available.

### COVID-19 infection

There has been a wide spectrum of neuro-ophthalmic manifestations of COVID-19 infection reported in the literature. Of the reports surveyed, there were 25 cases of newly diagnosed optic neuritis (16 unilateral, 9 bilateral), 10 of which were seropositive for MOG IgG, one demonstrated MOG positivity in the cerebrospinal fluid (CSF), and one seropositive for AQP4 IgG indicative of neuromyelitis optica spectrum disorder (NMOSD), 14 cases of idiopathic intracranial hypertension (also known as pseudotumour cerebri), three cases of posterior reversible encephalopathy syndrome, one case of myasthenia gravis, one case of occipital stroke, one case of orbital inflammation and optic perineuritis, one case of infarction of left optic nerve due to thrombosis of left internal carotid artery, one case of papillophlebitis, one case of acute macular neuroretinopathy, one case of non-arteritic anterior ischemic optic neuropathy.

The timing of onset of visual symptoms ranged from being concurrent with active COVID-19 infection to seven months following infection.

### Optic neuritis post COVID-19 infection

Both unilateral and bilateral presentations of optic neuritis have been reported at higher frequencies since the onset of the COVID-19 pandemic (Przybek-Skrzypecka et al., 2022). Of the 25 cases included in this review, the average time to symptomatic onset following infection is 32.8 days (range 0 – 210 days). All 25 cases with new-onset optic neuritis following or concurrent with COVID-19 infection had at least one MRI throughout their disease (**Supplementary Table 1**). A suspicion of multiple sclerosis (MS) was initially documented in seven cases, but further evaluation either through neuroimaging or lumbar puncture did not confirm a diagnosis of MS. Furthermore, 11 of the cases of optic neuritis demonstrated antibody positivity to myelin oligodendrocyte glycoprotein and one case was seropositive for aquaporin-4 (AQP4). High dose intravenous Methylprednisolone (IVMP) followed by oral steroid taper was administered in all patients except three [one of whom was suspected for having Vogt-Koyanagi-Harada disease due to concurrent severe intraocular inflammation (Benito-Pascual et al., 2020) and one in whom treatment was not mentioned (Borrego-Sanz et al., 2021)]. The mean visual acuity at presentation was 6/30 and mean visual acuity after treatment was 6/9. Visual acuity remained poor in one case despite prompt initiation of IVMP (Rodríguez-Rodríguez et al., 2021). Vision was also poor in another case, however IVMP was not promptly initiated and the patient was instead treated with topical and ocular corticosteroids due to concurrent panuveitis (François et al., 2021). Visual outcomes were not mentioned in three cases.

### MOG optic neuritis post COVID-19 infection

A subset of COVD-19 related optic neuritis demonstrated myelin oligodendrocyte glycoprotein antibody positivity. MOG antibody disease is an inflammatory central nervous system (CNS) disorder that manifests as unilateral or bilateral optic neuritis, with or without multi-focal demyelination and inflammation of the brain and spinal cord.

Ten of the patients in this review were seropositive for MOG IgG either concurrent with or following COVID-19 infection, with one further case demonstrating MOG positivity through CSF analysis. Seven cases were unilateral, four bilateral. On neuroimaging, eight cases had signal abnormalities of the optic nerve, four had additional lesions of the spinal cord, and two cases had lesions in the brain parenchyma. All 11 patients received IVMP, with two cases further requiring plasma exchange (PLEX) (Feizi et al., 2022; Johnsson et al., 2022). Mean visual acuity at presentation of the MOG IgG positive cohort was 6/19, which improved to a mean visual acuity of 6/6 following treatment.

### Idiopathic intracranial hypertension (IIH) post COVID-19 infection

There were 14 cases of newly diagnosed IIH identified in this review. The mean time of onset following COVID-19 infection is 8.7 days (range 0 to 42 days). Eleven of the 14 cases had neuroimaging with normal brain parenchyma, one further case was an 11-year-old girl initially diagnosed with multisystem inflammatory syndrome prior to IIH who had hyperintensities on FLAIR/T2 sequences and restricted diffusion in the splenium of the corpus callosum on MRI (Sofuoğlu et al., 2021). Neuroimaging was not mentioned in two cases. 13 of the 14 cases of IIH described in this review underwent a lumbar puncture with elevated opening pressure and normal CSF constituents, which fulfils the revised diagnostic criteria of IIH in adults and children (Friedman et al., 2013). The remaining case declined a lumbar puncture and was diagnosed based on clinical findings and neuroimaging.

### Myasthenia gravis post COVID-19 infection

Both generalised (Restivo et al., 2020) and ocular MG have been reported to be triggered by COVID-19 (Sriwastava et al., 2021). One case described in this review had a confirmed diagnosis of ocular MG through both positive acetylcholine receptor (AChR) antibodies and single fibre electromyography (EMG) (De Giglio et al., 2023). Another case of ocular MG concurrent with COVID-19 reported by Sriwastava et al. also demonstrated AChR antibody positivity and EMG findings consistent with a neuromuscular transmission deficit (Sriwastava et al., 2021). Three further cases were described by Restivo et. al, two of whom also reported systemic in addition to ocular symptoms of MG including general diplopia, ptosis, muscular fatigability, hypophonia, and dysphagia concurrent with COVID-19 and had their diagnosis confirmed through positive AChR antibody titres and EMG findings of postsynaptic deficit of neuromuscular transmission (Restivo et al., 2020).

### Posterior reversible encephalopathy syndrome post COVID-19 infection

A series of three cases of PRES were described in this review, with mean time of symptomatic onset being 20 days (range 9 to 39 days) following the development of COVID-19 symptoms (Hixon et al., 2021). All three cases presented with seizure-like activity, with two demonstrating visual field deficits on examination. All three cases had neuroimaging which showed T2/FLAIR hyperintensities spread throughout the brain parenchyma, two of which did not have corresponding diffusion restriction on diffusion-weighted imaging. Humphrey visual field (HVF) testing at six-month follow-up revealed a persistent left homonymous hemianopia for one patient and was declined by the other patient. Cerebrospinal fluid testing was not completed in all three patients.

### Long-term impact of COVID-19 infection

Two large population studies have proposed that COVID-19 increases the risks of developing neurological diseases. The first study utilised the US department of Veteran Affairs records to create a cohort of 154,068 patients with COVID-19, 5,638,795 contemporary controls and 5,859,621 historical controls (Xu et al., 2022). The burden of neurological complications was calculated to be the number of additional cases per 1000 persons at 12 months. They found that the hazard ratio (HR) of any neurologic sequelae in the post-acute phase of COVID-19 was 1.42 (95% confidence intervals 1.38, 1.47) and burden 70.69 (95% confidence interval 63.54, 78.01) additional sequelae per 1000 persons at 12 months. They investigated the risks of developing neurological disorders at 12 months following COVID-19 infection, and found an increased risk of a multitude of disorders including ischemic stroke [HR 1.50 (1.41, 1.61); burden 3.40 (2.75, 4.09)], haemorrhagic stroke [HR 2.19 (1.29, 5.62); burden 0.21 (0.11, 0.35)] cognition and memory disorders [HR 1.77 (1.68, 1.85); burden 10.07 (9.00, 11.20)], extrapyramidal movement disorders such as Parkinson-like disease [HR 1.50 (1.28, 1.7s; burden 0.89 (0.50, 1.34)], Guillain-Barré syndrome [HR 2.16 (1.40, 3.35); burden 0.11 (0.04, 0.22)], encephalitis and encephalopathy [HR 1.82 (1.16, 2.84); burden 0.07 (0.01, 0.16)] (Xu et al., 2022).

The second study is an analysis of two-year retrospective cohort studies extracted from TriNetX electronic health records network with a cohort of 1,284,437 propensity score matched patients recently infected with COVID-19 versus controls with another respiratory infection (Taquet et al., 2022). They found increased risks of cognitive deficit [HR 1.36 (1.33, 1.39)], dementia [HR 1.33 (1.26, 1.41)], intracranial haemorrhage [HR 1.09 (1.01, 1.18)], ischemic stroke [HR 1.11 (1.06, 1.17)], and epilepsy [HR 1.14 (1.09, 1.19)], whilst hazard ratios of encephalitis, GBS, nerve root, plexus disorder and parkinsonism were less than 1 (Taquet et al., 2022).

### COVID-19 vaccination

Vaccination campaigns are integral to the prevention of widespread infectious disease (Lotan et al., 2022). Development of vaccination campaigns drew from previous experiences in dealing with MERS and SARS-CoV, specifically the latter as SARS-CoV-2 and SARS-CoV are 79% genetically similar and both target the human Angiotensin Converting Enzyme 2 (hACE2) on the host cell (Kaur and Gupta, 2020). Post-vaccination neuro-ophthalmic manifestations were previously considered to be rare (Lotan et al., 2022), with optic neuritis following vaccination being the main complication reported (DeStefano et al., 2003; Stübgen, 2013; Karussis and Petrou, 2014; Cheng and Margo, 2022a).

As of 30^th^ March 2023, the World Health Organisation summarised that there are 183 vaccines in clinical development and 199 in pre-clinical development (WHO, 2023a). The 9 leading candidates are as follows: The Pfizer-BioNTech (BNT162b2) and the Moderna (mRNA-1273) messenger RNA (mRNA) vaccines, the AstraZeneca (AZD1222, ChAdOx1 nCoV-19, Vaxzevria), Janssen (Johnson & Johnson, Ad26.COV2.S), and Gamaleya (Sputnik V) non-replicating viral vector vaccines, the Sinovac (CoronaVac), Covaxin and Sinopharm (BBIBP-CorV) inactivated viral vaccines and the Novavax (NVX-CoV-2373) protein subunit vaccine (Kaur and Gupta, 2020; Li et al., 2021; Rawat et al., 2021; Hadj Hassine, 2022).

Safety data from phase 3 clinical trials have not highlighted neuro-ophthalmic diseases as a notable complication of the available COVID-19 vaccines (Palacios et al., 2020; Polack et al., 2020; Baden et al., 2021; Sadoff et al., 2021; Voysey et al., 2021). However, these trials are usually conducted over a limited time period and enrol no more than tens of thousands of participants, hence do not capture all the possible post-vaccination complications (Lotan et al., 2022).

Of the literature surveyed in this review, 31 cases of adverse neuro-ophthalmic events following COVID-19 vaccination were reported. 15 cases occurred following vaccination with Pfizer-BioNTech, nine cases following AstraZeneca, three cases following Moderna, two cases following Coronavac, and two cases following Sinopharm.

### Optic neuritis post COVID-19 vaccination

There were 27 cases of optic neuritis identified in this review, 17 had unilateral and 10 had bilateral involvement. Four cases were seropositive for MOG-IgG whilst two were seropositive for AQP4. 25 cases were of new-onset optic neuritis, whereas two cases had a history of optic neuritis; one had an episode of retrobulbar optic neuritis in 2014 and the other a history of multiple sclerosis with two previous episodes of optic neuritis (Shemer et al., 2023). The average time to symptom onset is 37 days (range 0.08 days (2 hours) to 180 days) following vaccination. Four cases were suspected of optic neuritis secondary to multiple sclerosis based on neuroimaging and CSF findings (Matsuo et al., 2023; Shemer et al., 2023). IVMP was commenced for all 27 cases, with three cases further requiring PLEX (Helmchen et al., 2022; Bhatti et al., 2023; Shemer et al., 2023). Improvement in visual function was noted for all except one case of unilateral NMO-associated optic neuritis where vision remained poor with optic atrophy observed following the second attack.

## Discussion

The incidence of neuro-ophthalmic sequelae following COVID-19 disease is far greater than that following vaccination against the virus. As of December 2023, there have been more than 770 million confirmed cases of COVID-19 (including nearly 7 million deaths) reported to WHO (2023b). At the same timepoint, 13 billion vaccine doses had been administered, with nearly 5.6 billion people vaccinated with a complete primary series. The 48 cases of adverse neuro-ophthalmic sequelae following infection and 31 cases following vaccination included in this review must be viewed in the context of the significant difference in denominator between these two groups.

It is difficult to conclude direct causation as opposed to correlation between COVID-19 and the neuro-ophthalmic sequelae, as the literature currently available primarily consists of individual case reports and small case series. Optic neuritis was the most frequently reported neuro-ophthalmic sequelae following COVID-19 infection and vaccination in this review, and prior to the onset of the pandemic is known to occur at a stable rate in the general population (Braithwaite et al., 2020). Optic neuritis can be associated with specific identifiable causes, such as multiple sclerosis, infection, or may be idiopathic, and the onset may seem to develop randomly without a trigger or cause. With neuro-ophthalmic conditions developing at a spontaneous low rate in the population at any time, many of the reported neurological sequelae following COVID-19 infection and vaccination in this review may have simply been unrelated coincidences.

In their multi-centre observational study, Zhao et al. analysed the rates of diagnosis of neuro-ophthalmic conditions as well as retinal detachment (RD) and acute angle closure glaucoma (AACG) (Zhao et al., 2024). They found that all conditions were diagnosed at higher rates following the introduction of COVID-19 vaccines compared to pre-COVID and pre-vaccine periods. The authors discuss that a likely explanation for the increased diagnostic rates following introduction of vaccinations is that patients tended to postpone seeking treatment even for acute ocular conditions such as RD and AACG during the pandemic, with clinic visit rates only noted to increase when vaccines became available to protect against the virus. Unlike neuro-ophthalmic conditions such as optic neuritis and cranial neuropathies, RD and AACG do not have plausible causal mechanisms in association with COVID-19 infection and vaccination, therefore the parallel fluctuation in diagnostic rates may point more towards correlation as opposed to causation. Nevertheless, the temporal association of the neuro-ophthalmic events occurring concurrent with or after SARS-CoV-2 infection or vaccination has led to the proposition of various causal pathomechanisms.

One theory of the pathogenesis of optic neuritis following COVID-19 infection is through direct viral inflammation of the optic nerve, subsequently causing myelin damage (Deane et al., 2021). This was demonstrated in mice inoculated with the mouse hepatitis virus MHV-A59 whereby upon isolation, the optic nerve sheaths and parenchyma were infiltrated with inflammatory cells (Shindler et al., 2008). It has been suggested that a similar mechanism may underlie optic neuritis as a result of COVID-19 infection as the mouse hepatitis virus shares a common genus with SARS-CoV-2 (Körner et al., 2020). An alternate theory is optic nerve ischemia secondary to a systemic state of hypercoagulability associated with SARS-CoV-2 infection. The increased thrombin generation and endothelial inflammation results in a hypoxic state which may induce an ischemic optic neuropathy (Deane et al., 2021). However, ischemia is less likely to be reversible, therefore the observation that most cases of optic neuritis concurrent with or following COVID-19 infection demonstrated improvement after treatment detracts from the likelihood of ischemia being the underlying causal mechanism.

Eleven of the 25 cases of new-onset optic neuritis in this review demonstrated MOG antibody positivity. It has been hypothesised that much like other viral infections, the aberrant post-infectious immune response following COVID-19 triggers the onset of a para-infectious demyelinating process (Tenembaum et al., 2007; Bosello et al., 2023). One suggested mechanism is through molecular mimicry, where viral antigens induce a pathological immune response against CNS antigens including antibodies against MOG (Tisdale et al., 2021; Bosello et al., 2023). However, it has also been pointed out that despite auto-antibodies targeting MOG, molecular mimicry is unlikely the cause due to lack of homology between the COVID-19 viral protein and the MOG protein (Johnsson et al., 2022). Instead, loss of self-tolerance in the context of an activated adaptive immune system is suggested to be culprit (Wesselingh and Wesselingh, 2023).

There are many cases in the literature of new-onset IIH following COVID-19 infection, with a limited consideration of how CSF hydrodynamics is affected by COVID-19 infection. Under physiological conditions, the choroid plexus secretes CSF (Wang et al., 2022) and is a known site of blood brain barrier breakdown in COVID-19 (Fullard et al., 2021; Yang et al., 2021). SARS-CoV-2 has tropism for the choroid plexus epithelium, meninges, and brain vasculature as the SARS-CoV-2 entry proteins ACE2 and TMPRSS2 are both expressed at these sites, which has been suggested as the underlying mechanism of dysregulated CSF hydrodynamics seen in COVID-19 (Yang et al., 2021). Endothelial dysfunction in COVID-19 also impairs CSF absorption via the arachnoid villi and astrocytic foot processes surrounding cerebral vasculature (Wang et al., 2022). Additionally, COVID-19 leads to a systemic state of hyperviscosity and hypercoagulability which increases venous pressure and induces formation of cerebral venous sinus thromboses, also leading to increased intracranial pressure (Al-Mufti et al., 2021; Mukharesh et al., 2022).

The pathogenesis of myasthenia gravis following COVID-19 is under investigation (Sriwastava et al., 2021). It is known that a systemic state of inflammation such as that following SARS-CoV-2 infection reduces the availability of AChRs at the postsynaptic neuromuscular junction (Koneczny and Herbst, 2019). Additionally, an external virus induces antibody production, which evokes an immune response that subsequently cross-reacts with AChRs as a result of molecular resemblance (Sriwastava et al., 2021). The affinity of COVID-19 for the ACE2 receptor found on multiple organs including the brain, lungs, and heart leads to the formation of autoantibodies and subsequently a downstream cascade involving proinflammatory cytokines, chemokines, depletion of B and T cells, and increased interleukins and TNF-a (Baig et al., 2020). This increases the risk of a cross-reactive autoimmune attack against the body’s own receptors (Baig et al., 2020). The treatment target is to improve symptoms, control antibody production and reduce disease severity, and typically encompasses pyridostigmine and immunosuppressive agents (Sriwastava et al., 2021).

PRES occurs due to cerebral oedema secondary to endothelial dysfunction that presents as visual disturbance, headache, seizures and altered consciousness (Triplett et al., 2022). SARS-CoV-2 has affinity for the viral receptor ACE2, which is also expressed at the capillary endothelium. The inflammatory nature of COVID-19 infection leading to vascular endothelial injury is suggested to be the causal mechanism of PRES development. The literature of PRES following COVID-19 infection present with similar symptoms to the three cases described in this review including altered mental status, seizure activity, visual impairment and visual agnosia (Franceschi et al., 2020; Kaya et al., 2020; Kishfy et al., 2020; Hixon et al., 2021). Neuroimaging in these three cases is also comparative with that described in the literature of T2/FLAIR hyperintensities diffusely distributed in the cerebral white matter without corresponding diffusion restriction on DWI (Hixon et al., 2021). The limiting factor is that none of the three cases underwent cerebrospinal fluid testing, hence direct infection resulting in the development of PRES cannot be excluded despite its uncommon occurrence.

Concerns for neurological complications of the vaccines began arising in 2020 when two patients developed transverse myelitis following the AstraZeneca vaccine (Goss et al., 2021). Serious systemic complications have been reported after vaccination, such as cerebral venous thrombosis as well as specific immune-mediated conditions such as myocarditis, pericarditis and thrombocytopenia (Chen et al., 2022). Most adverse neurological sequelae occurred after AstraZeneca vaccinations, followed by Pfizer-BioNTech and Moderna vaccines (Sriwastava et al., 2022). However the rates of each vaccination vary widely, for example as of November 2023, the total vaccine doses administered in the European Union were Pfizer-BioNTech 654 million, Moderna 155 million, AstraZeneca 67 million, Johnson & Johnson 19 million, Sinopharm/Beijing 2 million, Sputnik V 2 million (Mathieu et al., 2021). In this review, Pfizer-BioNTech was associated with nearly half of the neuro-ophthalmic complications reported, followed by AstraZeneca and Moderna, but it is clearly difficult to compare the rates of complications between vaccines when the absolute risks are so low and the numbers of vaccine doses administered varied so widely.

Optic neuritis was the most common complication following COVID-19 vaccination in this review. Optic neuritis following vaccine administration has previously been reported for other anti-viral vaccines, including but not limited to measles/mumps/rubella, influenza, rabies, tetanus/diphtheria/pertussis, hepatitis B and *Herpes Zoster* (Stübgen, 2013; Cheng and Margo, 2022a; Bhatti et al., 2023). There is no definitive causal relationship between vaccination and optic neuritis onset (Bhatti et al., 2023), although propositions of the etiopathogenesis include reaction to a vaccine component (e.g. protein or preparation adjuvant), molecular mimicry, production of proinflammatory cytokines, and/or activation of the innate and adaptive immune system (Bhatti et al., 2023). Nevertheless, reports of serious neurological sequelae following COVID-19 vaccination are still considered rare, especially in comparison with that caused by COVID-19 infection which has been found to be 617-fold higher in an analysis from the vaccine adverse event reporting system (VAERS) (Frontera et al., 2022).

## Conclusion

As we approach the end of the SARS-CoV-2 pandemic, there is an increased awareness of the neuro-ophthalmic complications brought on by viral insult. The various COVID-19 vaccines that are currently available are part of the global effort to protect the most vulnerable of the human population. The incidence of neuro-ophthalmic consequences following infection with COVID-19 is hundred-folds higher and associated with more harrowing systemic effects than that following vaccination against the virus. Several proposed causal mechanisms are discussed in this review, but larger studies are required to make any definitive conclusions. As we approach the post-COVID era, neuro-ophthalmic presentations should nevertheless always necessitate careful exploration of COVID-19 infection history and vaccination status.

## Author contributions

JS: Writing – original draft. HD: Writing – review & editing.

## Supplementary material

Supplementary Table 1 Summary of case reports of neuro-ophthalmic events following COVID-19 infection and vaccination. Key: MRI, magnetic resonance imaging; MRV, magnetic resonance venography; FLAIR, fluid attenuated inversion recovery; MOG, myelin oligodendrocyte glycoprotein; AQP4, aquaporin-4; CT, computed tomography; IV, intravenous; IVMP, intravenous Methylprednisolone; RAPD, relative afferent pupillary defect; w/wo, with/without; PLEX, plasma exchange; CF, counting fingers; HVF, Humphrey visual field; BP, blood pressure; RE, right eye; LE, left eye; R, right; L, left; VA, visual acuity; CSF, cerebrospinal fluid; IPH, intraparietal haemorrhage; DWI, diffusion-weighted imaging; OCT, optical coherence tomography; CNS, central nervous system.
