# Supplementary material for: A review of neuro-ophthalmic sequelae following COVID-19 infection and vaccination
Source: Front Cell Infect Microbiol. 2024 Jan 17;14:1345683. doi: 10.3389/fcimb.2024.1345683 (PMC10827868; doi:10.3389/fcimb.2024.1345683)
Supplement: Supplementary Table 1 — Summary of case reports of neuro-ophthalmic events following COVID-19 infection and vaccination. Key: MRI, magnetic resonance imaging; MRV, magnetic resonance venography; FLAIR, fluid attenuated inversion recovery; MOG, myelin oligodendrocyte glycoprotein; AQP4, aquaporin-4; CT, computed tomography; IV, intravenous; IVMP, intravenous Methylprednisolone; RAPD, relative afferent pupillary defect; w/wo, with/without; PLEX, plasma exchange; CF, counting fingers; HVF, Humphrey visual field; BP, blood pressure; RE, right eye; LE, left eye; R, right; L, left; VA, visual acuity; CSF, cerebrospinal fluid; IPH, intraparietal haemorrhage; DWI, diffusion-weighted imaging; OCT, optical coherence tomography; CNS, central nervous system. [file Table_1.docx]

Supplementary Material

| **Authors** | **Gender** | **Age (year)** | **Presenting complaint** | **Time following COVID-19 infection or vaccination (days)** | **Diagnosis** | **Ocular examination findings** | **MRI findings** | **Additional features** |
| --- | --- | --- | --- | --- | --- | --- | --- | --- |
| Caudill and Wolin (62) | Female | 72 | Bilateral visual loss | 10 days following infection | Optic neuritis with bilateral involvement | Reduced visual acuity to light perception in both eyes. Minimally reactive pupils. Disc oedema in the right eye and disc atrophy in the left eye | MRI brain with and without gadolinium: No acute ischemic process with no intracranial contrast enhancement  MRI orbits: Enhancement of orbital segments of both optic nerves | Not associated with MOG or AQP4  Treated with 6-day high-dose IV Methylprednisolone followed by oral prednisone taper  Recovery of vision in each eye with full visual fields following treatment |
| Bosello et. al (42) | Female | 74 | Right eye visual loss and pain increasing with eye movements | 60 days following infection | Optic neuritis with unilateral involvement | Reduced visual acuity in right eye) with right RAPD. 12 hours later the vision in right eye declined, which prompted urgent initiation of IVMP | MRI brain and orbits with gadolinium revealed a FLAIR and T2 signal alteration at the middle portion of the retrobulbar intra-orbital segment of the right optic nerve, and slight perineural enhancement around the nerve | Serum MOG antibody positive  Treated with 3-day intravenous Methylprednisolone 500mg followed by Prednisone 50mg with slow tapering. Intravenous immunoglobulins (2 g/kg) over 5 days were administered with substantial improvement  After 2 weeks, the patient reported complete resolution of pain and vision improved in the right eye. |
| Feizi et. al (10) | Male | 41 | Left visual blurriness, retro-orbital pain and headache | 210 days following infection | Optic neuritis with unilateral involvement | Reduced in left eye of 20/70 (6/21) with left RAPD. | Initial MRI of spine and brain w/wo contrast suggested LETM and post-infectious transverse myelitis. Repeat MRI brain showed a new T2-FLAIR lesion of the left corona radiata and right parietal subcortical white matter.  Subsequent MRI brain and orbits w/wo contrast 6 months later showed pre-chiasmatic (intracanalicular) enhancement of left optic nerve | Serum MOG antibody positive  Treated with IVMP 1g/day for 5 days initially. 6 months later IVMP 1g/day restarted for 3 days to treat optic neuritis with 3 further cycles of PLEX therapy. Monthly IVIg therapy for suspected post-COVID MOGAD optic neuritis  Significant improvement in visual acuity almost returning to baseline at follow-up |
| Johnsson et. al (11) | Male | 20 | Reduced vision, headache, photo- and phonophobia, left hemianesthesia, paraparesis | 56 days following infection | Optic neuritis with bilateral involvement | Bilateral reduced visual acuity (not specified), reduced colour vision and optic disc oedema | MRI of spinal cord showed pronounced medullary T2 lesions from T9 to conus medullaris and small focal lesions at T7 and C6 levels, and slight contrast enhancement at T9-T11. | Serum MOG antibody positive  Treated with PLEX on consecutive days followed by IVMP 1g/day for 3 days. Oral Prednisolone tapered to 10mg daily and 15mg Methotrexate weekly.  Residual symptoms of moderate paraparesis following treatment |
| Johnsson et. al (11) | Male | 29 | Bilateral visual impairment (not specified) and frontal headache | 15 days following infection | Optic neuritis with bilateral involvement | Papillitis | MRI brain normal | Serum MOG antibody positive  Treated with IVMP 1g/day for 3 days followed by oral Prednisolone tapered to 10mg daily  Vision returned to normal within a month |
| Johnsson et. al (11) | Female | 60 | Right eye pain | 54 days following infection | Optic neuritis with unilateral involvement | Reduced visual acuity to 20/100 (6/30) and papillitis in the right eye | MRI showed swelling and contrast enhancement in the right optic nerve, no significant pathology in cerebral parenchyma or spinal cord | Serum MOG antibody positive  Treated with IVMP 1g/day for 3 days followed by oral Prednisolone with slow tapering  Recovery not mentioned |
| Ide et. al (63) | Female | 24 | Reduced vision in left eye | 21 days following infection | Optic neuritis with unilateral involvement | Reduced visual acuity to 0.6 (6/10) and optic nerve oedema in left eye | Initial brain CT revealed mild swelling of the left optic nerve. Contrast-enhanced MRI brain showed faint T2 extensive lesions. MRI spinal cord showed scattered mottled or linear T2 high-signal lesions. | Serum MOG antibody positive  Treated with 2 courses of Methylprednisolone pulse therapy (1g/day) for 5 days then switched to oral Prednisolone  Vision in left eye spontaneously improved prior to commencement of steroid therapy. No recurrence of systemic symptoms four months after discharge from hospital |
| Gilardi et. al (64) | Male | 56 | Left eye pain worsened by eye movements and blurred vision | 15 days following infection | Optic neuritis with unilateral involvement | Reduced visual acuity and colour vision in left eye with left RAPD. Left optic disc swelling with peripapillary haemorrhages | MRI brain w/wo contrast showed diffuse left optic nerve hyperintensity in T2/FLAIR without gadolinium enhancement. No abnormalities found in spine | Serum MOG antibody positive  Treated with IVMP 1g/day for 3 days followed by oral Prednisone (1mg/kg/day) and subsequent tapering  Complete visual recovery 10 days after starting therapy |
| Kivanany et. al (65) | Female | 35 | Reduced vision in the right eye, pain with eye movements and worsening headaches | 49 days following infection | Optic neuritis with bilateral involvement | Reduced visual acuity in both eyes with right more so than left. Right RAPD. Bilateral optic disc oedema. Subsequent worsening headaches and new vision loss in right eye and painful eye movements 10 days later | MRI brain and orbits with contrast and venogram showed empty sella and bilateral papilledema. No venous sinus thrombosis. MRI ~10 days later showed right optic nerve enhancement and non-enhancing central cord abnormality at T4 extending to T7-T8 | Serum AQP4 antibody positive  Empirically treated with intravenous Aciclovir, Cefepime, and Vancomycin and PO Acetazolamide 500mg BD. 10 days later treated with 5 days of IVMP and 5 cycles of plasma exchange  No significant visual improvement |
| Micieli and Yu (66) | Male | 31 | Visual loss in right eye and right eye pain worsened with eye movements. | 12 days following infection | Optic neuritis with unilateral involvement | Reduced visual acuity in right eye (CF, ~6/150) and right RAPD. Mild right optic disc oedema. | MRI or the brain/orbits with contrast showed increased T2-weighted signal intensity, enlargement, and enhancement of the intraorbital/intracanalicular segments of the right optic nerve and sheath. Concurrent high T2/FLAIR signal intensity along lateral margins of pons without enhancement | Not associated with MOG or AQP4  Treated with IVMP 1g/day for 5 days followed by Prednisone 1mg/kg daily  At three months following onset of symptoms, improved visual acuity with a normal HVF 24-2 |
| Duran and Aykaç (67) | Female | 30 | Reduced vision in left eye | 4 days following infection | Optic neuritis with unilateral involvement | Reduced visual acuity in left eye. Mild left disc oedema with left RAPD | MRI brain and spine normal.  MRI orbits showed linear enhancement areas located in the left retrobulbar region | Not associated with MOG or AQP4  Treated with 3 days of intravenous steroid therapy followed by oral steroid (1mg/kg)  After 2 weeks, visual acuity in left eye was improved with marked reduction in disc oedema and improvement in HVF |
| Deane et. al (39) | Female | 21 | Blurry vision in the left eye | Concurrent with infection | Optic neuritis with unilateral involvement | Reduced visual acuity of hand movements in left eye. Left optic disc elevation 360 degrees with obscuration of vessels | MRI brain showed non-enhancing T2/FLAIR subcortical white-matter hyperintensities, mainly in the frontal lobes, that may be indicative of demyelinating disease. Concurrent abnormal T2 FLAIR hyperdense signals in the left optic nerve reflecting acute optic neuritis | No antibody association  Treated with Methylprednisolone and Remdesivir for 5 days. Discharged with oral steroids  Vision returned to normal with no visual field deficits and normal fundoscopy at 1 month following initial presentation |
| Sawalha et. al (68) | Male | 44 | Bilateral eye pain and vision loss, right initially then progressing to left | 14 days following infection | Optic neuritis with bilateral involvement | Reduced visual acuity in both eyes right > left with right RAPD. | MRI brain showed enhancement in the right more than the left optic nerve suggestive of optic neuritis | MOG antibodies detected in cerebrospinal fluid  Treated with Methylprednisolone 1g/day for 5 days. Discharged on tapering Prednisone over 4 weeks  Complete restoration of vision in the left eye with remarkable but not complete vision recovery in the right eye |
| Rodriguez-Rodriguez et. al (8) | Female | 55 | Headache and left eye pain exacerbated by eye movements | Concurrent with infection | Optic neuritis with unilateral involvement | Reduced visual acuity in left eye and left RAPD | MRI brain normal. MRI orbits showed mild increased thickness and signal in the left optic nerve | Not associated with MOG or AQP4  Treated with IVMP 1g/day for 5 days followed by oral Prednisone taper  Ocular pain decreased 3 months later however vision did not improve at 1 month follow-up |
| Jossy et. al (69) | Male | 16 | Sudden loss of vision in the left eye, headache and pain on eye movements | 14 days following infection | Optic neuritis with unilateral involvement | Reduced vision in the left eye with left RAPD. | MRI brain and spine normal. MRI orbits showed hyperintensity in the intraorbital and intracanalicular part of the left optic nerve | Not associated with MOG or AQP4  Treated with IVMP 1g/day for 3 days followed by oral steroids 1mg/kg x 11 days and tapering over the next 3 days  Improvement in vision noted after IVMP |
| Jossy et. al (69) | Male | 35 | Sudden loss of vision in the left eye with pain on eye movements | 180 days following infection | Optic neuritis with unilateral involvement | Reduced vision in left eye with left RAPD. Left oedematous disc with blurred margins and peripapillary oedema | MRI brain, spinal cord and orbits all normal | Not associated with MOG or AQP4  Treated with IVMP 1g/day for 3 days followed by oral steroids 1mg/kg x 11 days and tapering over the next 3 days  Vision in LE improved at 2 months follow-up |
| Jossy et. al (69) | Male | 38 | Sudden reduction of vision in left eye with pain on eye movements | 42 days following infection | Optic neuritis with unilateral involvement | Reduced vision in the left eye to hand movements with left RAPD | MRI brain and spinal cord normal. MRI of the orbits showed hyperintense lesions along both optic nerves suggestive of demyelination | Serum MOG antibody positive  Treated with IVMP 1g/day for 3 days followed by oral steroids 1mg/kg x 11 days and tapering over the next 3 days  Vision improved in LE at 2 months follow-up |
| Benito-Pascual et. al (6) | Female | 60 | Left ocular pain, blurred vision and redness | Concurrent with infection | Optic neuritis with unilateral involvement | Reduced vision in left eye with left RAPD. Left panuveitis and focal posterior synechiae. Left vitritis with optic nerve swelling and peripapillary subretinal fluid and peripapillary choroidal folds | Neuroimaging normal | Not associated with MOG or AQP4  Probable Vogt-Koyanagi-Harada syndrome was suspected so treatment with oral Prednisone, hourly steroid drops, and mydriatics three times a day was commenced  Visual acuity improved in the left eye following treatment |
| Sainath et. al (70) | Female | 56 | Sudden bilateral visual loss with painful eye movements | 14 days following infection | Optic neuritis with bilateral involvement | Reduced visual acuity and defective colour vision in both eyes to counting fingers. Painful bilateral extraocular movement on superior and lateral gaze | MRI of the brain and orbit revealed swelling of the right retrobulbar intraorbital segment of the optic nerve with a high T2 signal | Not associated with MOG or AQP4  Treated with intravenous pulsed Methylprednisolone 250mg every 6 hours/day x 3 days followed with oral Methylprednisolone 1mg/kg/weight once daily x 11 days  Improved within 1 week in both eyes and defective colour vision. HVF 30-2 on recovery showed a paracentral scotoma in both eyes |
| Kogure et. al (71) | Male | 47 | Left eye pain and superior visual field defect | Concurrent with infection | Optic neuritis with unilateral involvement | Reduced visual acuity in left with left RAPD. Pain on extraocular movements in left eye. | MRI brain and orbits with gadolinium: Post-contrast T1-weighted fat-suppressed MRI showed bilateral (but left-dominant) uniform enhancement along optic nerve sheaths | Serum MOG antibody positive  Treated with Methylprednisolone 1g/day for 3 days followed by oral Prednisolone taper  Pain with eye movements immediately relieved after starting steroid therapy. Vision improved with disappearance of left RAPD at 2-week follow-up |
| Sinha et. al (72) | Male | 13 | Sudden painless visual blurring | Concurrent with infection | Optic neuritis with bilateral involvement | Reduced visual acuity and sluggish pupillary reactions in both eyes. Bilateral hyperaemic and elevated optic discs with blurring of margins and obliteration of optic cup | MRI brain and orbits normal | Not associated with MOG or AQP4  Treated with IV antibiotics, anticoagulant, IVIg and Methylprednisolone  Near total restoration of visual functions with improvement in vision of both eyes following treatment |
| Zhou et. al (73) | Male | 26 | Bilateral pain with eye movements and subacute sequential vision loss initially affecting left then right eye | Concurrent with infection | Optic neuritis with bilateral involvement | Reduced visual acuity in both eyes with right > left and right RAPD (right eye hand movements so 6/190 and left eye 20/250 so 6/75) . Bilateral disc oedema and venous congestion with right retina perivenous haemorrhage | MRI brain and orbits w/wo contrast revealed uniform enhancement and thickening of both optic nerves extending from the globe to their intracranial pre-chiasmal segments. One small non-enhancing, non-specific periventricular T2 hyperintensity was present adjacent to the occipital horn of the right lateral ventricle.  MRI spinal cord w/wo contrast noted patchy T2 hyperintensities in the lower cervical and upper thoracic spinal cord associated with mild central thickening and gadolinium enhancement | Serum MOG antibody positivity  Treated with IVMP 1g/day for 5 days followed by oral Prednisone taper.  VA improved in each eye with complete resolution of disc oedema at 3-week follow-up |
| Borrego-Sanz et. al (7) | Female | 66 | Painless loss of vision in the left eye | 40 days following infection | Optic neuritis with unilateral involvement | Reduced vision in the left eye to hand movements with left RAPD. Marked left optic nerve pallor and large cupping with narrowing of retinal arterioles | MRI orbits showed a discrete signal enhancement in the left optic nerve suggestive of optic neuropathy without any parenchymal ischemic alteration | Not associated with MOG or AQP4  Treatment not mentioned  Recovery not mentioned |
| François et. al (9) | Female | Late 50’s | Blurry vision, redness and pain with eye movements in right eye | 2 days following infection | Optic neuritis with unilateral involvement | Reduced visual acuity, colour vision and contrast in right eye with right RAPD. Right non-granulomatous retro-descemetic precipitates and anterior chamber inflammation. Right optic disc oedema, peripapillary haemorrhages, mild vitreous inflammation and retinal vessel narrowing. | MRI brain with fine optic nerve cuts unremarkable | Not associated with MOG or AQP4  Initially treated with oral and topical corticosteroids for presumed non-infectious ocular inflammation  Visual acuity remained poor in right eye at 1.5-month follow-up. Fundoscopy revealed severe papillary atrophy in right eye |
| Sharma et. al (74) | Female | 22 | Visual blurring and perceiving an absolute scotoma in inferior field of right eye | 6 days following infection | Optic neuritis with unilateral involvement | Reduced visual acuity in right eye of with right RAPD. Delayed but full colour vision and inferior visual field defect in right eye. Nasal, superior and inferior blurring of disc margin with tortuosity and dilatation of vessels in right eye. | MRI with gadolinium of brain and orbits normal | Not investigated for MOG or AQP4 antibody association n  Treated with IVMP 1g/day over 3 days and discharged on oral Prednisolone  Resolution of disc oedema in the inferior margin noted prior to discharge. Did not present for face-to-face follow-up, however she reported subjective resolution of the inferior scotoma on telephone follow-up |
| Rajabi et. al (75) | Female | 13 | Anorexia, nausea, vomiting, fever, headache, and diplopia | 20 days following infection | Idiopathic intracranial hypertension | Esotropia of left eye with bilateral abduction limitation of eyes consistent with 6^th^ nerve palsy and papilledema | Normal MRI brain and CT orbit. MRV was normal without venous thrombosis | Lumbar puncture opening pressure of 40 cm H_2_O. Normal CSF composition |
| Mukharesh et. al (48) | Female | 22 | Headaches, blurred vision, diplopia, pulse-synchronous tinnitus, and vision changes | Concurrent with infection | Idiopathic intracranial hypertension | Frisen grade 5 papillledema with macular exudates | Not mentioned | Lumbar puncture opening pressure of 50 cm H_2_O |
| Mukharesh et. al (48) | Female | 30 | Daily non-migrainous headaches, pulse-synchronous tinnitus and vision changes | 2 days following infection | Idiopathic intracranial hypertension | Frisen grade 2 papilledema | MRI brain showed partially empty sella | Patient declined lumbar puncture |
| Mukharesh et. al (48) | Female | 34 | Headaches and blurry vision in both eyes | 14 days following infection | Idiopathic intracranial hypertension | Frisen grade 4 papilledema with peripapillary haemorrhages | MRI brain demonstrated signs of increased intracranial pressure without cerebral venous sinus thrombosis | Lumbar puncture opening pressure 53 cm H_2_O |
| Mukharesh et. al (48) | Female | 36 | Headaches and blurry vision in both eyes | Concurrent with infection | Idiopathic intracranial hypertension | Frisen grade 2 papilledema | MRI/MRV of the brain revealed neuroimaging stigmata of pseudotumour cerebri syndrome | Lumbar puncture opening pressure 28 cm H_2_O |
| Mukharesh et. al (48) | Female | 25 | Worsening headaches, bilateral dimness of vision, and pulse-synchronous tinnitus | 7 days following infection | Idiopathic intracranial hypertension | Reduced visual acuity with left more significant than right, dyschromatopsia, left RAPD and Frisen grade 5 papilledema | Not mentioned | Lumbar puncture opening pressure of 60 cm H_2_O |
| Mukharesh et. al (48) | Female | 51 | Worsening headaches with blurred vision | Concurrent with infection | Idiopathic intracranial hypertension | Frisen grade 2 papilledema and constricted visual fields | MRI/MRV of the brain was concerning for possible small cortical vein thrombosis but the large venous sinuses were patent | Lumbar puncture opening pressure 26 cm H_2_O |
| Mukharesh et. al (48) | Female | 13 | Dimness and blurriness of vision in right eye. Headaches worsening in a recumbent position | 5 days following infection | Idiopathic intracranial hypertension | Reduced visual acuity with Frisen grade 2 papilledema | MRI/MRV normal | Lumbar puncture opening pressure 38 cm H_2_O |
| Mukharesh et. al (48) | Female | 33 | Progressive headaches and transient visual obscurations | Concurrent with infection | Idiopathic intracranial hypertension | Frisen grade 1 papilledema and mild nasal field deficits with left greater than right eye | MRI/MRV showed an empty sella | Lumbar puncture opening pressure 30 cm H_2_O |
| Balendra et. al (76) | Female | 29 | Headaches with visual blurring and pulsatile tinnitus | 42 days following infection | Idiopathic intracranial hypertension | Reduced visual acuity and colour vision bilateral, Frisen grade 5 papilledema | MRI brain with contrast showed dilated optic nerve sheaths | Lumbar puncture opening pressure > 80 cm CSF with normal constituents |
| Balendra et. al (76) | Female | 16 | Double vision worsening with right gaze and right sided headaches | 9 days following infection | Idiopathic intracranial hypertension | Right abducens nerve palsy without papilledema with subsequent left abducens nerve palsy | MRI brain with venography demonstrates signs consistent with raised ICP without evidence of venous sinus thrombosis | Lumbar puncture opening pressure 39 cm CSF with normal constituents |
| Khalid and Micieli (77) | Female | 22 | Headaches with retrobulbar pain | 7 days following infection | Idiopathic intracranial hypertension | Bilateral optic disc oedema with peripapillary wrinkles in right eye | MRI and MRV brain showed flattening of the posterior globes, increased CSF space around the optic nerves, and distal transverse sinus stenosis | Lumbar puncture opening pressure of 37 cm H_2_O and normal CSF constituents |
| [Sofuoğlu](https://pubmed.ncbi.nlm.nih.gov/?term=Sofuo%C4%9Flu%20A%C4%B0%5BAuthor%5D) et. al (12) | Female | 11 | Headache and diplopia | 10 days following infection | Idiopathic intracranial hypertension | Left abducens nerve paralysis and grade 3 papilledema | MRI brain showed hyperintensities on T2/FLAIR sequences, restricted diffusion in the splenium of the corpus callosum, and optic nerve head protrusion with globe flattening | Lumbar puncture CSF analysis shows acellular constituents. Opening pressure not documented |
| Verkuil et. al (78) | Female | 14 | Esotropia | 6 days following infection | Idiopathic intracranial hypertension | Right abducens nerve palsy, papilledema, and left disc haemorrhage | MRI and MRV brain revealed abnormalities consistent with raised intracranial pressure | Lumbar puncture opening pressure 36 cm H_2_O with 2 white blood cells/µL, normal glucose and protein. |
| Hixon et. al (17) | Female | 69 | Seizure-like jerking of left face, arm, and leg, and left gaze deviation | 12 days following infection | Posterior reversible encephalopathy syndrome | Normal visual acuity. Left homonymous hemianopia on visual field testing | MRI brain showed non-enhancing, bilateral T2-weighted/FLAIR hyperintensities in the parietal and occipital cortex and subcortical white matter. DWI showed no corresponding reduced diffusion | Maximum BP of 180/90 mmHg noted prior to seizure. Strict BP protocol initiated with Hydralazine  Follow-up MRI at 2-months following discharge showed resolution of T2/FLAIR hyperintensities  Persistent left homonymous hemianopia at 6-month follow-up |
| Hixon et. al (17) | Female | 55 | Bilateral upper and lower extremity weakness, visual loss, vertigo, and seizure | 9 days following infection | Posterior reversible encephalopathy syndrome | Normal visual acuity with no fundus abnormalities. Left sided visual deficit | MRI brain showed diffuse T2/FLAIR hyperintense oedema of the occipital, parietal, and posterior frontal white matter consistent with PRES with a very minimal superior convexity subarachnoid haemorrhage, and without corresponding diffusion restriction on DWI | Seizures treated with Levetiracetam 1500mg and Lorazepam 4mg initially  BP during the seizure event was 178/88. Maintained on strict BP control with Nicardipine  Declined follow-up visual field testing and MRI  General Optometry examination revealed normal visual acuity and no posterior fundus abnormalities |
| Hixon et. al (17) | Male | 65 | Bilateral tonic-clonic seizure | 39 days following infection | Posterior reversible encephalopathy syndrome | Not reported | CT showed a new right sided intraparenchymal haemorrhage. MRI revealed symmetric T2/FLAIR white matter hyperintensities in the posterior frontal lobes and frontoparietal junctions consistent with PRES, with corresponding diffusion restriction only at the site of the IPH on DWI | Medical background of pyoderma gangrenosum on immunosuppressive medication (Prednisone, Infliximab, and Adalimumab)  Single transient BP of 163/119 at 12 hours before the seizure  Started on Levetiracetam 1000mg twice daily with no seizure recurrence  CT and MRI at 1-month post-discharge showed resolution of bilateral PRES findings. Repeat neurological examination showed no visual abnormalities |
| De Giglio et. al (16) | Male | 74 | Rapidly worsening diplopia | 60 days following infection | Myasthenia gravis and Graves disease | 3mm left exophthalmos with lid retraction on downgaze. Left adduction deficit with fatiguability. Bilateral impairment of elevation and infraduction with variable left ptosis worsening with sustained upgaze | MRI of the brain was normal while coronal study of orbits showed thickening of inferior, medial and superior rectus muscles in both eyes. | Myasthenia gravis diagnosed based on positive acetylcholine receptor antibodies and single fibre electromyography findings of altered jitter values and decremental response on repeated stimulation  Commenced on Prednisone, Pyrostigmine, and Tapazole  Complete and stable recovery of ocular motility at 6-month follow-up |
| Dinkin et. al (79) | Male | 56 | Headaches above right eyebrow worse with eye movements and transient “cloudy white out” in right eye lasting from 3-15 minutes each | Concurrent with infection | Orbital inflammation and optic perineuritis | Reduced visual acuity in right eye, 2+ RAPD which resolved with symptoms. Normal anterior and posterior segment examination | MRI orbits showed perineural enhancement with extension in the orbital fat of the right eye consistent with orbital inflammation and optic perineuritis | 3-week Prednisone taper was prescribed  Repeat MRI at 2-month follow-up showed some improvement but persistent perineuritis despite clinical improvement  Clinical examination at 3-month follow-up show improved visual acuity |
| Tavakoli (80) | Male | 50 | Vision loss in left eye. | 21 days following infection | Infarction of left optic nerve due to thrombosis of left internal carotid artery | Left RAPD with unremarkable eye movements and fundus examination | CT angiography consistent with occlusion of the cervical and intracranial portion of the left internal carotid artery. MRI brain showed scattered acute infarcts in the distribution of the left middle cerebral artery. DWI showed restricted diffusion along the intraorbital portion of the left optic nerve | No treatment mentioned.  Six weeks later, visual function was unchanged however the left optic disc was pale |
| Insausti-García et. al (81) | Male | 40 | Painless decrease in visual sensitivity in left eye. | 42 days following infection | Papillophlebitis | Normal visual acuities with no RAPD. Severe inflammation of left optic nerve head with retinal venous vasodilatation and tortuosity, cotton-wool spots, and moderate superficial haemorrhages in all four quadrants | Unremarkable | One week after diagnosis, visual acuity decreased in the left eye due to macular oedema.  Sustained-release dexamethasone implant was injected  A marked reduction in macular and papillary oedema and progressive visual recovery was noted two weeks following injeciton |
| Virgo and Mohamed (82) | Male | 32 | Right eye paracentral scotoma | 16 days following infection | Acute macular neuroretinopathy | Normal visual acuity and fundus examination | Not mentioned | Changes on infrared reflectance and OCT correlated with the location of the scotoma with a focal area of faint outer plexiform layer hyper-reflective change and disruption of the interdigitation zone  Treatment not mentioned |
| Sitaula et. al (83) | Female | 60 | Acute painless visual loss in left eye associated with floaters | Concurrent with infection | Non-arteritic anterior ischemic optic neuropathy | Reduced left visual acuity with grade 2 RAPD and sectoral disc pallor with disc oedema. Left hemifield defect with red and green colour deficiency. Reduction in left contrast sensitivity | MRI of the brain and orbits with contrast normal | OCT showed increased retinal nerve fibre layer thickness and HVF showed an inferior altitudinal defect in the LE  Commenced on Aspirin  Visual acuity had improved in the left eye at 1-month follow-up, however the inferior hemifield defect persisted on confrontational visual field testing |
| Kang et. al (84) | Female | 42 | Ocular pain and visual impairment on left side | 7 days following vaccination  Pfizer-BioNTech – 3^rd^ dose | Optic neuritis with unilateral involvement | Reduced visual acuity and left RAPD | MRI orbits showed slight enlargement and T2 high signal intensities with contrast enhancement in the left optic nerve. MRI brain normal. MRI spine revealed an asymptomatic, short-segment, non-enhancing T2 hyperintense lesion in the spinal cord at C6 | Serum MOG antibody positive  Medical history of pre-diagnosed systemic lupus erythematosus  Treated with IVMP 1g daily for 5 days  Visual acuity improved following treatment |
| Arnao et. al (85) | Female | Middle-aged | Reduced bilateral visual acuity, headache and painful blurred vision worsened by eye movement | 14 days following vaccination  AstraZeneca – 1^st^ dose | Retrobulbar optic neuritis with bilateral involvement | Reduced visual acuity, reduced colour vision and ring scotomas bilaterally. No optic nerve abnormalities | Brain CT and CT venogram – no abnormalities detected | Not associated with MOG or AQP4  Treated with IVMP 1g/day for 5 days  Patient’s symptoms greatly improved following treatment |
| Roy et. al (86) | Female | 27 | Blurring of vision in left eye and mild periocular pain | 9 days following vaccination  AstraZeneca – 1^st^ dose | Optic neuritis with unilateral involvement | Reduced left visual acuity, left RAPD and colour desaturation. Diffuse swelling of left optic nerve head | MRI brain and orbit showed T2 enhancement of left optic nerve head just behind the disc | Not associated with MOG or AQP4  Treated with IVMP pulse therapy started for 3 days followed by oral steroid  Vision in left eye improved following treatment |
| Roy et. al (86) | Female | 48 | Gradual painless reduction in vision in left eye | 5 days following vaccination  AstraZeneca – 2^nd^ dose | Optic neuritis with unilateral involvement | Reduced left visual acuity with RAPD. Swollen left optic disc with blurred margins | MRI brain and orbit within normal limits | Not associated with MOG or AQP4  Treated with IVMP pulse therapy  Vision in left eye significantly improved following treatment |
| Roy et. al (86) | Male | 40 | Sudden blurring of vision in both eyes | 12 days following vaccination  AstraZeneca – 1^st^ dose | Optic neuritis with bilateral involvement | Reduced bilateral visual acuity with bilaterally blurred and swollen optic disc margin | Not completed prior to treatment | Not associated with MOG or AQP4  Treated with steroid therapy  Vision improved in both eyes following treatment |
| Leber et. al (87) | Female | 32 | Headache, low visual acuity, pain on movement of left eye and loss of temporal visual field in left eye | 0.5 days (12 hours) following vaccination  CoronaVac – 2^nd^ dose | Optic neuritis with bilateral involvement | Reduced visual acuity in left eye, L RAPD, bilateral disc swelling worse in left eye | MRI brain no signs of demyelinating disease such as MS or CNS tumour. MRI orbit showed hyper signal on T2/FLAIR and gadolinium enhancement in the anterior portion of both optic nerves, more intense in the left eye | Serum MOG antibody positive  Concurrent acute thyroiditis  Treated with IVMP 1g/day for 5 days followed by tapering corticosteroid therapy  Vision improved following treatment |
| Matsuo et. al (36) | Female | 74 | Reduced visual acuity | 120 days following vaccination  Moderna – 4^th^ dose | Optic neuritis with bilateral involvement | Bilateral swollen optic nerves and left isolated retinal periphlebitis along inferior vascular arcade | MRI showed several non-continuous multifocal lesions with contrast enhancement in the cervical and upper thoracic spinal cord without lesions in the brain | Not associated with MOG or AQP4  Neuroimaging and CSF results suggest possible multiple sclerosis  Concurrent hypophysitis with diabetes insipidus. Medical history of myelodysplastic syndrome diagnosed 10 years previously  Treated with 2 courses of steroid pulse therapy with methylprednisolone 1g daily for 3 days  Vision improved in both eyes following treatment |
| Bhatti et. al (37) | Female | 43 | Flashing lights and blurred vision in right eye associated with painful eye movement which progressed to the left eye | 6 days following vaccination  Pfizer-BioNTech – 1^st^ dose | Optic neuritis with bilateral involvement | Bilateral reduced visual acuity of counting fingers with initial R RAPD documented. Normal fundal examination | MRI brain and orbits with contrast showed enhancement of both optic nerve sheaths | Not associated with MOG or AQP4  Medical history of type 2 diabetes mellitus, arterial hypertension, secondary hypothyroidism following radioactive iodine treatment for Graves disease and migraines  Treated with 3 consecutive days of IVMP at 1g/day and 7 treatments of plasma exchange  Vision improved in both eyes following treatment |
| Zhang et. al (88) | Female | 58 | Bilateral eye pain worsened by eye movement | 0.08 days (2 hours) following vaccination  Pfizer-BioNTech – booster vaccination, dose unspecified | Optic neuritis with unilateral involvement | Mild left hypertropia on right gaze, -3 deficit in supraduction on the right, and right eye pain with upgaze. Normal visual acuity. Mild nasal and inferior optic nerve rim elevation | MRI brain and orbits showed right optic nerve enhancement and non-specific foci of high intensity in the periventricular and centrum semiovale regions of the brain. MRI spinal cord normal | Not associated with MOG or AQP4  COVID-19 infection three weeks prior. Nil significant ocular or medical history  Treated with 3-day course of IVMP 250mg every 6 hours with 11-day oral steroid taper  Eye pain and motility deficits completely improved following treatment |
| Shemer et. al (35) | Male | 38 | Reduced vision and eye pain on left side | 135 days following vaccination  Pfizer-BioNTech – 2^nd^ dose | Retrobulbar optic neuritis with unilateral involvement | Mildly reduced left visual acuity, pain with eye movement, L RAPD and colour desaturation in left eye. Normal fundoscopy | MRI brain and orbits showed a prechiasmatic, hyperintense lesion on T2, enhancing with gadolinium of the left optic nerve | Not associated with MOG or AQP4  Previous retrobulbar optic neuritis in 2014  Treated with IVMP for 3 days followed by oral steroids  Improvement in both vision and perimetry in the left eye following treatment |
| Shemer et. al (35) | Female | 29 | Headache, visual loss in right eye followed by appearance of grey spots on lower visual fields | 180 days following vaccination  Pfizer-BioNTech – dose unspecified | Optic neuritis with unilateral involvement | Reduced right visual acuity and colour desaturation with R RAPD. Normal fundoscopy | MRI brain and orbits shows diffuse abnormal signal from the right optic nerve without enhancement and abnormal signal foci in the white matter and corpus callosum, compatible with multiple sclerosis | Not associated with MOG or AQP4  Neuroimaging suggestive of multiple sclerosis  Treated with IVMP for 3 days followed by 5-day course of plasmapheresis  Mild improvement in VA with residual positive RAPD and colour desaturation in the right eye following treatment |
| Shemer et. al (35) | Male | 54 | Right visual loss | 180 days following vaccination  Pfizer-BioNTech – 2^nd^ dose | Optic neuritis with unilateral involvement | Reduced right visual acuity to counting fingers. Pain with eye movement, R RAPD, and colour desaturation in right eye. Blurred supero-nasal disc margin in right eye | MRI Brain and orbit showed abnormal signal in a short segment of the right optic nerve | Not associated with MOG or AQP4  Treated with IVMP 1g/day for 3 days followed by oral Prednisone  Improved vision in right eye following treatment |
| Shemer et. al (35) | Female | 38 | Headache and left eye pain | 60 days following vaccination  Pfizer-BioNTech – 2^nd^ dose | Optic neuritis with unilateral involvement | Pain induced by eye movement and trace L RAPD. Mildly reduced vision in left eye. Normal fundoscopy | MRI brain and orbits showed thickening of the left optic nerve | Serum MOG antibody positive  Treated with IVMP for 5 days followed by oral Prednisone  Excellent functional recovery following treatment |
| Shemer et. al (35) | Female | 42 | Visual loss in left eye and pain on eye movement | 21 days following vaccination  Pfizer-BioNTech – 2^nd^ dose | Optic neuritis with unilateral involvement | Pain induced by eye movement in left eye and L RAPD. Normal visual acuity and fundoscopy | MRI showed enhancement in the prechiasmatic optic nerve segment in the left eye, without cerebral lesions | Not associated with MOG or AQP4  Presumed associated with multiple sclerosis. Family history of sibling with multiple sclerosis  Treated with IVMP 1g/day for 5 days followed by oral Prednisone  One week later, VA, RAPD and colour vision remained impaired in the left eye whilst a mild improvement noted on automated perimetry |
| Shemer et. al (35) | Female | 45 | Blurred vision in right eye | 42 days following vaccination  Pfizer-BioNTech – 2^nd^ dose | Optic neuritis with unilateral involvement | Reduced right visual acuity with R RAPD and colour desaturation in right eye. Eye movements induced moderate pain. Normal fundoscopy | MRI found abnormal signals in the white matter characteristic of active multiple sclerosis | Not associated with MOG or AQP4  Neuroimaging suggestive of multiple sclerosis. Medical history of multiple sclerosis with 2 previous episodes of optic neuritis  Treated with IVMP for 5 days followed by oral Prednisone  Significant improvement in VA and visual field with a negative RAPD following treatment |
| Shemer et. al (35) | Male | 14 | Occipital and frontal headaches with blurred vision and photophobia | 63 days following vaccination  Pfizer-BioNTech – 2^nd^ dose | Optic neuritis with bilateral involvement | L RAPD and colour desaturation in left eye with reduced visual acuity in right eye. Swollen right optic disc with vascular lining | MRI found abnormal signal in the left intraorbital optic nerve with enhancement | Not associated with MOG or AQP4  Positive Epstein-Barr nuclear antigen, which may suggest a post-infectious aetiology  Treated with IVMP  Good visual function recovery noted following treatment |
| Shukla et. al (89) | Female | 56 | Progressive painless vision loss in left eye followed by sudden, painless vision loss in the right eye | 12 days following vaccination  AstraZeneca – 1^st^ dose | Optic neuritis with bilateral involvement | NPL in both eyes with bilateral sluggish pupils. Pale discs with distinct margins | MRI brain and orbit showed increase T2 signal intensities in bilateral optic nerves | Not associated with MOG or AQP4  Concurrent sarcoidosis supported by histological and radiological results. Nil medical history  Treated with 3 days of pulse steroids followed by 1g/kg Prednisolone  Visual acuity improved to 6/60 bilaterally following treatment |
| Lee (90) | Female | 28 | Right visual decline and pain on ocular movement | 14 days following vaccination  AstraZeneca – 1^st^ dose | Optic neuritis with unilateral involvement | Reduced visual acuity in right eye with R RAPD. Mild right colour desaturation. Normal fundoscopy | MRI orbit no evidence of gadolinium enhancement of optic nerve on T2 images with fat suppression or high T2 signal | Not associated with MOG or AQP4  Treatment with IVMP 1g/day for 3 days with standard oral Prednisolone tapering for 3 weeks  Vision in right eye following treatment |
| Wang et al (91) | Female | 21 | Blurred vision in right eye with pain on ocular movement | 21 days after vaccination  Sinopharm - 2^nd^ dose | Optic neuritis with unilateral involvement | Reduced visual acuity in right eye with R RAPD. Blurred margins of right optic disc with congestion and oedema | MRI orbit no significant abnormalities | Not associated with MOG or AQP4  Treated with IVMP 800mg/day for 3 days and then oral Prednisone with gradual dose reduction  Vision improved in the right eye following treatment |
| Wang et al (91) | Female | 38 | Blurred vision in right eye | 21 days following vaccination  Sinopharm – 1^st^ dose | Optic neuritis with unilateral involvement | Reduced visual acuity in right eye with R RAPD. Blurred borders of right optic disc with congestion and oedema | CT orbits showed hypointense thickening of the right optic nerve with normal cranial CT | Serum MOG antibody positive  Treated with IVMP 1g/day for 3 days then oral Prednisone with gradual dose reduction  Vision improved in the right eye following treatment |
| Tasci et. al (92) | Male | 32 | Blurred vision and retrobulbar pain in right eye | 14 days following vaccination  CoronaVac – 1^st^ dose | Optic neuritis with unilateral involvement | Reduced visual acuity in right eye with R RAPD. Right colour desaturation. Mild swelling and blurry nasal optic margin in right eye | MRI of brain, orbits and spine w/wo contrast revealed uniform contrast enhancement and thickening of the intraorbital part of the right optic nerve but no lesions elsewhere | Serum AQP4 antibody positive  Diagnosis of gastric neuroendocrine tumour during treatment, leading to delay of immunosuppressive treatment  Treated with IVMP 1g/day for 5 days followed by 1mg/kg/day of oral corticosteroids  Vision initially improved in the right eye. 2 weeks later the patient presented with sudden right visual loss and was further treated with IVMP 1g/day for 5 days followed by oral prednisone 1mg/kg/day for 6 weeks.  Vision remained poor in the right eye following the second episode of visual loss |
| Helmchen et. al (38) | Female | 40 | Blurring of vision rapidly developing into binocular blindness | 14 days following vaccination  AstraZeneca – 1^st^ dose | Optic neuritis with bilateral involvement | No ocular examination documented. | MRI brain showed increased signal intensity at the chiasm and part of the adjacent optic nerves and tracts with mild contrast enhancement of the optic chiasma. MRI spine showed increased longitudinal centrally located signal intensities throughout the thoracic myelin indicating a myelitis | Associated with longitudinally extensive myelitis (LETM) resembling NMO-negative NMOSD  Longstanding history of relapsing-remitting multiple sclerosis  Treated with IVMP 2g/day, plasmapheresis and immunoadsorption  Improved visual acuity but unchanged paraplegia following treatment |
| Nagaratnam et. al (93) | Female | 36 | Right sided headache, blurred vision and photophobia in the right eye | 14 days following vaccination  AstraZeneca – 1^st^ dose | Optic neuritis with bilateral involvement | Normal visual acuity initially. 2 days later bilateral visual impairment and colour desaturation with painful eye movements. No RAPD. Normal fundoscopy | 3 T MRI brain on day 2 showed multiple T2/FLAIR hyperintense lesions in the subcortical white matter, posterior limb of bilateral internal capsules, pons, and left cerebral middle cerebellar peduncle. No abnormal signal in optic nerves and no evidence of demyelinating disease. MRI on day 17 showed abnormal signal and enhancement of both optic nerves but more prominent on the left | Associated with acute disseminated encephalomyelitis (ADEM) based on neuroimaging and cerebrospinal fluid analysis  Not associated with MOG or AQP4  Nil significant medical history. Mother has multiple sclerosis  Treated with IVMP 1g/day for 3 days without tapering oral steroids.  Initial marked improvement in vision, however re-presented with worsening vision in both eyes and further 3 doses of IVMP 1g/day followed by oral Prednisolone 50mg daily with a tapering plan  Visual acuity improved in both eyes following treatment |
| Pirani et. al (94) | Female | 31 | Visual loss in right eye | 6 days following vaccination  Pfizer-BioNTech – 1^st^ dose | Retrobulbar optic neuritis with unilateral involvement | Reduced visual acuity in right eye. Right optic disc with peripapillary atrophy and blurred margins | T1-weighted MRI of brain and orbits with fat suppression sequences and post gadolinium contrast showed enhancement of the retrobulbar right optic nerve near the optic foramen | Not associated with MOG or AQP4  Medical history of ankylosing spondylitis  Treated with IVMP 1g/day for 5 days followed by tapering doses of oral Prednisone over the next 10 days  Vision improved in the right eye following treatment |
| Pirani et. al (94) | Female | 46 | Visual loss in right eye | 8 days following vaccination  Pfizer-BioNTech – 1^st^ dose | Retrobulbar optic neuritis with unilateral involvement | Reduced visual acuity in the right eye. Anterior and posterior examination normal | MRI confirmed diagnosis of retrobulbar optic neuritis | Not associated with MOG or AQP4  Medical history of Hashimoto thyroiditis and a viral myocarditis  Treated with IVMP 1g/day for 5 days followed by tapering doses of oral Prednisone over the next 10 days  Vision improved in the right eye following treatment |
| Elnahry et. al (95) | Female | 69 | Sudden onset blurry vision in both eyes | 16 days following vaccination  Pfizer-BioNTech – 2^nd^ dose | Papillitis with bilateral involvement from post-vaccination central nervous system inflammatory syndrome | Reduced visual acuity in right eye of counting fingers with a R RAPD. Bilateral optic nerve head oedema, right greater than left, associated with peripapillary haemorrhages | MRI of brain, orbits, and spine normal | Not associated with MOG or AQP4  Concurrent neuroretinitis supported by OCT and fundus fluorescein angiography findings. Medical history of hypertension, type 2 diabetes, and cutaneous T-cell lymphoma in remission  Treated with IVMP 1g/day for 5 days  Vision and visual field stabilised following treatment |
| Elnahry et. al (95) | Female | 32 | Progressive blurring of vision in left eye | 6 days following vaccination  AstraZeneca – 1^st^ dose | Optic neuritis with unilateral involvement | Reduced visual acuity in left eye with colour desaturation and left RAPD. Mild left optic disc hyperaemia and swelling. | MRI of the brain, orbit and cervical spine showed left optic nerve enhancement | Not associated with MOG or AQP4  Treated with IVMP 1g/day for 3 days followed by 1mg/kg/day of oral Prednisone, gradually tapered  Vision improved in the left eye following treatment |
| Jarius et. al (96) | Male | 67 | Reduction in vision and colour desaturation in left eye associated with temporal headache and pain on eye movement | 10 days following vaccination  Pfizer-BioNTech – 3^rd^ dose | MOG encephalitis | No posterior segment abnormality | MRI showed swelling and contrast enhancement of the anterior part of the left optic nerve with no brain or spinal cord lesions | Serum MOG antibody positive  Medical history of arterial hypertension and benign prostate hyperplasia  Treated with IVMP 1g/day for 3 days followed by oral tapering of Methylprednisolone over 44 days  Complete recovery except for residual phosphenes in the dark following treatment |
| Karam et. al (97) | Male | 43 | Horizontal painless diplopia | 4 days following vaccination  Pfizer-BioNTech – 2^nd^ dose | Isolated left 6^th^ nerve palsy | 10 dioptre left esotropia in primary gaze increasing to 16 dioptres on left gaze and reducing to 6 dioptres on right gaze. Remaining neurological and ophthalmic exam normal | MRI brain showed focal enlargement of the root exit zone and the cisternal portion of the left 6^th^ nerve with post-gadolinium enhancement. Further MRI 8 weeks after presentation showed residual minimal enhancement of the 6^th^ nerve | No treatment documented  Resolved within 5 weeks |
| Mohamed et. al (98) | Female | 71 | Loss of colour vision and decreased visual acuity in right eye | 150 days following vaccination  Moderna – 2^nd^ dose | Dysthyroid optic neuropathy | Reduction of colour vision bilaterally, right greater than left, with eye pain, redness, lid oedema and erythema, diplopia, and proptosis | Orbital CT showed enlargement of the extraocular muscles bilaterally and mild bilateral exophthalmos | 40-year medical history of hypothyroidism controlled on Levothyroxine  Treated with IVMP 1g/day for 2 days followed by IV Teprotumumab every 3 weeks  Return of colour vision in both eyes and improvement of proptosis and periorbital oedema after her third Teprotumumab infusion |
| Chuang et. al (99) | Male | 45 | Left sided headache, left eye pain with progressive left ptosis, reduced vision and binocular diplopia | 7 days following vaccination  Moderna – dose unspecified | Tolosa-Hunt syndrome supported by neuroimaging | Left ptosis with L RAPD with complete ophthalmoplegia | CT head showed hyperattenuation of the left cavernous sinus. Contrast-enhanced MRI of the brain and orbits and MRA/MRV of the head showed bilateral perineural enhancement surrounding the optic nerve sheaths, left greater than right. Also an ill-defined enhancement in the left orbital apex extending into the cavernous sinus suggestive of an inflammatory vs infective process | Initially treated with broad-spectrum antibiotics, which were discontinued after infection ruled out. Subsequently treated with IVMP 1g/day for 3 days followed by an oral steroid regimen  Improvement in cranial nerve deficits |

**Table 1** – summary of case reports of neuro-ophthalmic events following COVID-19 infection and vaccination

Key: MRI, magnetic resonance imaging; MRV, magnetic resonance venography; FLAIR, fluid attenuated inversion recovery; MOG, myelin oligodendrocyte glycoprotein; AQP4, aquaporin-4; CT, computed tomography; IV, intravenous; IVMP, intravenous Methylprednisolone; RAPD, relative afferent pupillary defect; w/wo, with/without; PLEX, plasma exchange; CF, counting fingers; HVF, Humphrey visual field; BP, blood pressure; RE, right eye; LE, left eye; R, right; L, left; VA, visual acuity; CSF, cerebrospinal fluid; IPH, intraparietal haemorrhage; DWI, diffusion-weighted imaging; OCT, optical coherence tomography; CNS, central nervous system

**References**

65. Caudill GB, Wolin MJ. Myelin oligodendrocyte glycoprotein and neuromyelitis optica/aquaporin-4 antibody negative COVID-19-associated optic neuritis. J Neuroophthalmol. 2023;43(1):e1-e2.

66. Ide T, Kawanami T, Eriguchi M, Hara H. SARS-CoV-2-related myelin oligodendrocyte glycoprotein antibody-associated disease: A case report and literature review. Intern Med. 2022;61(8):1253-8.

67. Gilardi M, Cortese A, Ferraro E, Rispoli M, Sadun R, Altavista MC, et al. MOG-IgG positive optic neuritis after SARS-CoV-2 infection. Eur J Ophthalmol. 2023;33(5):Np87-np90.

68. Kivanany PB, Raviskanthan S, Mortensen PW, Lee AG. Antiaquaporin 4-related optic neuritis and myelitis post-COVID-19 infection. J Neuroophthalmol. 2022;42(4):e571-e3.

69. Micieli JA, Yu CW. Optic neuritis associated with SARS-CoV-2 B.1.1.7 variant of concern. Can J Neurol Sci. 2022;49(4):591-2.

70. Duran M, Aykaç S. Optic neuritis after COVID-19 infection: A case report. J Fr Ophtalmol. 2023;46(1):e4-e7.

71. Sawalha K, Adeodokun S, Kamoga GR. COVID-19-induced acute bilateral optic neuritis. J Investig Med High Impact Case Rep. 2020;8:2324709620976018.

72. Jossy A, Jacob N, Sarkar S, Gokhale T, Kaliaperumal S, Deb AK. COVID-19-associated optic neuritis - A case series and review of literature. Indian J Ophthalmol. 2022;70(1):310-6.

73. Sainath D, Paul A, Krishnagopal S, Kumar A. Acute bilateral retrobulbar optic neuritis - an atypical sequela of COVID-19. Indian J Ophthalmol. 2021;69(12):3761-4.

74. Kogure C, Kikushima W, Fukuda Y, Hasebe Y, Takahashi T, Shibuya T, et al. Myelin oligodendrocyte glycoprotein antibody-associated optic neuritis in a COVID-19 patient: A case report. Medicine (Baltimore). 2021;100(19):e25865.

75. Sinha A, Dwivedi D, Dwivedi A, Bajaj N. Optic neuritis as a presenting symptom of post-COVID-19 multisystem inflammatory syndrome in children (MIS-C). Indian J Pediatr. 2021;88(12):1269.

76. Zhou S, Jones-Lopez EC, Soneji DJ, Azevedo CJ, Patel VR. Myelin oligodendrocyte glycoprotein antibody-associated optic neuritis and myelitis in COVID-19. J Neuroophthalmol. 2020;40(3):398-402.

77. Sharma A, Kudchadkar US, Shirodkar R, Usgaonkar UPS, Naik A. Unilateral inferior altitudinal visual field defect related to COVID-19. Indian J Ophthalmol. 2021;69(4):989-91.

78. Rajabi MT, Rafizadeh SM, Aghajani AH, Pirzadeh M. Idiopathic intracranial hypertension as a neurological manifestation of COVID-19: A case report. J Fr Ophtalmol. 2022;45(7):e303-e5.

79. Balendra R, North M, Kumar G, Qutab S, Raouf HA, Delamont SS, et al. Raised intracranial pressure (pseudotumour cerebri) associated with severe acute respiratory syndrome coronavirus 2. J Neuroophthalmol. 2022;42(2):e459-e62.

80. Khalid MF, Micieli JA. Idiopathic intracranial hypertension associated with SARS-CoV-2 B.1.1.7 variant of concern. Can J Neurol Sci. 2022;49(3):472-3.

81. Verkuil LD, Liu GT, Brahma VL, Avery RA. Pseudotumor cerebri syndrome associated with MIS-C: a case report. Lancet. 2020;396(10250):532.

82. Dinkin M, Feinberg E, Oliveira C, Tsai J. Orbital inflammation with optic perineuritis in association with COVID-19. J Neuroophthalmol. 2022;42(1):e300-e1.

83. Tavakoli M, Sotoudeh H, Rezaei A, Saadatpour Z, Vaphides MS, Kline LB. Optic nerve infarction in a patient with Coronavirus disease 2019. J Neuroophthalmol. 2022;42(1):e347-e8.

84. Insausti-García A, Reche-Sainz JA, Ruiz-Arranz C, López Vázquez Á, Ferro-Osuna M. Papillophlebitis in a COVID-19 patient: Inflammation and hypercoagulable state. Eur J Ophthalmol. 2022;32(1):Np168-np72.

85. Virgo J, Mohamed M. Paracentral acute middle maculopathy and acute macular neuroretinopathy following SARS-CoV-2 infection. Eye (Lond). 2020;34(12):2352-3.

86. Sitaula S, Poudel A, Gajurel BP. Non-arteritic anterior ischemic optic neuropathy in COVID-19 infection - A case report. Am J Ophthalmol Case Rep. 2022;27:101684.

87. Kang M, Kim S, Park JS, Seok HY. Myelin oligodendrocyte glycoprotein antibody-associated optic neuritis following third dose of BNT162b2 COVID-19 vaccine in a patient with systemic lupus erythematosus. Neurol Sci. 2023;44(7):2247-9.

88. Arnao V, Maimone MB, Perini V, Giudice GL, Cottone S. Bilateral optic neuritis after COVID vaccination. Neurol Sci. 2022;43(5):2965-6.

89. Roy M, Chandra A, Roy S, Shrotriya C. Optic neuritis following COVID-19 vaccination: Coincidence or side-effect? - A case series. Indian J Ophthalmol. 2022;70(2):679-83.

90. Leber HM, Sant'Ana L, Konichi da Silva NR, Raio MC, Mazzeo T, Endo CM, et al. Acute thyroiditis and bilateral optic neuritis following SARS-CoV-2 vaccination with CoronaVac: A case report. Ocul Immunol Inflamm. 2021;29(6):1200-6.

91. Zhang J, Joiner D, Zhang C. Hyperacute optic neuritis in a patient with COVID-19 infection and vaccination: a case report. BMC Ophthalmol. 2023;23(1):80.

92. Shukla AK, Peter A, Bhargava JK, Arya V, Gupta MK, Yadav N, et al. Sarcoidosis presenting as bilateral optic neuritis after ChAdOx1 nCoV-19 vaccination. Monaldi Arch Chest Dis. 2022;93(1).

93. Lee WA. COVID-19 vaccine-associated optic neuritis. Qjm. 2022;115(10):683-5.

94. Wang J, Huang S, Yu Z, Zhang S, Hou G, Xu S. Unilateral optic neuritis after vaccination against the coronavirus disease: two case reports. Doc Ophthalmol. 2022;145(1):65-70.

95. Yıldız Tascı Y, Nalcacoglu P, Gumusyayla S, Vural G, Toklu Y, Yesılırmak N. Aquaporin-4 protein antibody-associated optic neuritis related to neuroendocrine tumor after receiving an inactive COVID-19 vaccine. Indian J Ophthalmol. 2022;70(5):1828-31.

96. Nagaratnam SA, Ferdi AC, Leaney J, Lee RLK, Hwang YT, Heard R. Acute disseminated encephalomyelitis with bilateral optic neuritis following ChAdOx1 COVID-19 vaccination. BMC Neurol. 2022;22(1):54.

97. Pirani V, Pelliccioni P, Carpenè MJ, Nicolai M, Barbotti F, Franceschi A, et al. Optic neuritis following COVID-19 vaccination: Do autoimmune diseases play a role? Eur J Ophthalmol. 2023;33(4):Np46-np50.

98. Elnahry AG, Asal ZB, Shaikh N, Dennett K, Abd Elmohsen MN, Elnahry GA, et al. Optic neuropathy after COVID-19 vaccination: a report of two cases. Int J Neurosci. 2023;133(8):901-7.

99. Jarius S, Bieber N, Haas J, Wildemann B. MOG encephalomyelitis after vaccination against severe acute respiratory syndrome coronavirus type 2 (SARS-CoV-2): case report and comprehensive review of the literature. J Neurol. 2022;269(10):5198-212.

100. Karam EZ, Ríos Macias P, Chahin G, Kattah JC. Inflammatory sixth nerve palsy post-COVID-19 vaccination: Magnetic resonance imaging findings. Neuroophthalmology. 2022;46(5):314-8.

101. Mohamed A, Tzoulis P, Kossler AL, Dosiou C. New onset or deterioration of thyroid eye disease after mRNA SARS-CoV-2 vaccines: Report of 2 cases and literature review. J Clin Endocrinol Metab. 2022;108(4):979-85.

102. Chuang TY, Burda K, Teklemariam E, Athar K. Tolosa-Hunt syndrome presenting after COVID-19 vaccination. Cureus. 2021;13(7):e16791.
